# Cognitive Sarcopenia: Prevalence and the Risk for Mortality and Healthy Aging in the KORA‐Age Study

**DOI:** 10.1002/jcsm.70201

**Published:** 2026-01-25

**Authors:** Marie‐Theres Huemer, Barbara Thorand, Eva Grill, Lars Schwettmann, Annette Peters

**Affiliations:** ^1^ Institute of Epidemiology Helmholtz Zentrum München, German Research Center for Environmental Health (GmbH) Neuherberg Germany; ^2^ Department of Epidemiology Harvard T.H. Chan School of Public Health Boston USA; ^3^ Institute for Medical Information Processing, Biometry and Epidemiology, Medical Faculty Ludwig‐Maximilians‐Universität München Munich Germany; ^4^ German Center for Vertigo and Balance Disorders, DSGZ, Faculty of Medicine Ludwig‐Maximilians Universität Munich (LMU) Munich Germany; ^5^ Institute of Health Economics and Health Care Management Helmholtz Zentrum München, German Research Center for Environmental Health (GmbH) Neuherberg Germany; ^6^ Department of Health Services Research, School of Medicine and Health Sciences Carl von Ossietzky University of Oldenburg Oldenburg Germany

**Keywords:** activities of daily living, cognitive impairment, cognitive sarcopenia, mortality, nursing care, sarcopenia

## Abstract

**Background:**

Cognitive sarcopenia, defined by this study as the co‐existence of sarcopenia and cognitive impairment, has been frequently reported in older adults, while we hypothesize that the co‐existence increases the risk for adverse outcomes in the older general population.

**Methods:**

This study included 1055 participants aged 65–93 years from the population‐based cohort Cooperative Health Research in the Region Augsburg (KORA)‐Age (2008/9). At baseline, probable sarcopenia (i.e., low grip strength) and confirmed sarcopenia (i.e., probable sarcopenia plus low muscle mass) were defined according to the European Working Group on Sarcopenia in Older People (EWGSOP) 2018 consensus. Cognitive impairment was derived from the modified telephone interview for cognitive status or a proxy interview with relatives/caregivers when participants had severe physical/mental impairment. Cognitive probable sarcopenia was defined as having both probable sarcopenia and cognitive impairment; cognitive confirmed sarcopenia as both confirmed sarcopenia and cognitive impairment. Isolated probable sarcopenia and isolated cognitive impairment refer to individuals with only one of the diseases. Mortality was assessed using death certificates over 12 years (553 deaths [52.4%]). Adverse outcomes were assessed in 2012 and 2016 during telephone interviews. Covariate‐adjusted logistic and Cox regression models estimated the associations with adverse outcomes and mortality, respectively.

**Results:**

Almost 50% of older adults with probable sarcopenia had cognitive impairment, whereas among older adults without probable sarcopenia, only 20% had cognitive impairment. A total of 8.1% of the study population had cognitive probable sarcopenia, while 3.3% had cognitive confirmed sarcopenia. Muscle mass was not [OR (95% CI): 0.92 (0.70–1.20)], while grip strength [0.73 (0.57–0.94)], gait speed [0.66 (0.54–0.80)], and Timed Up and Go time [1.51 (1.27–1.82)] were associated with cognitive impairment. Participants with cognitive probable sarcopenia had an increased risk of all‐cause mortality [HR (95% CI): 1.95 (1.41–2.70)], cardiovascular disease mortality [1.64 (1.02–2.64)], and coronary heart disease mortality [2.10 (1.03–4.27)] after 12 years, and activities of daily living disability [OR (95% CI): 6.12 (2.33–16.06)] and requiring nursing care after 3 years [4.77 (1.47–14.63)]. Individuals with isolated probable sarcopenia or isolated cognitive impairment had either lower or no risk for those outcomes.

**Conclusions:**

Since life expectancy and relevant healthcare have not advanced considerably since study baseline, we expect that these results are relevant today. The high prevalence of cognitive impairment in older adults with probable sarcopenia and the increased risk of cognitive sarcopenia on lifespan and independence endorse screening for cognitive impairment in older adults with probable sarcopenia by EWGSOP 2018 and support exploring intervention studies targeting both diseases simultaneously.

## 1 Introduction

Aging is a major common risk factor for many related and interlinked diseases, which initiated the concept to target multiple chronic diseases during aging simultaneously [1]. To determine which diseases simply accumulate during aging and which diseases add an additional risk, disentangling the diseases' joint and individual pathological load on health‐ and lifespan is crucial. One of the key aging‐related diseases severely impacting the health and abilities of older people [2] is sarcopenia, a muscle disease characterized by low muscle strength (probable sarcopenia), plus low muscle mass (confirmed sarcopenia) and low function (severe sarcopenia) according to the European Working Group on Sarcopenia in Older People (EWGSOP) [3]. In addition to the severe physical deterioration, sarcopenia has been frequently observed to co‐exist with cognitive impairment. However, a standardized term for this co‐existence emphasizing its relevance and facilitating comparability between studies is currently missing. Therefore, in accordance with the term *cognitive frailty*—‘simultaneous presence of both physical frailty and cognitive impairment’ [4]—this study describes the co‐existence of both sarcopenia and cognitive impairment as *cognitive sarcopenia*. To differentiate between sarcopenia defined by only low muscle strength (probable) versus low muscle strength plus low muscle mass (confirmed), *cognitive probable sarcopenia* represents having both probable sarcopenia and cognitive impairment, while *cognitive confirmed sarcopenia* indicates having both confirmed sarcopenia and cognitive impairment.

Since there is plenty of evidence on the cross‐sectional association between sarcopenia and cognitive impairment, as demonstrated by multiple meta‐analyses [5, 6, 7, 8, 9], and both diseases develop during aging, a linked pathophysiology has been considered. Suggested links of sarcopenia influencing cognition include abnormal synthesis of myokines [10], brain atrophy and inflammation, whereas cognitive impairment has been suspected to promote sarcopenia through reduced physical activity and motoric skills [11]. We therefore hypothesize that their co‐existence increases the risk for mortality and other adverse outcomes in older adults. Initial studies observed an association of cognitive sarcopenia (defined by different parameters) with a higher mortality risk in former hospital patients in Italy after 12 months [12] and in US‐American older adults after a median follow‐up of 48 months [13], while it remains unclear if cognitive sarcopenia is linked to mortality during longer follow‐up times in the general population. Cognitive probable sarcopenia was further observed to increase the odds of activities of daily living (ADL) disability compared to any of the individual diseases in older American adults [14]. However, it remains unknown if cognitive sarcopenia increases the risk of cause‐specific mortality and major adverse health outcomes relevant for older adults including hospitalization, requiring nursing care, falls and multimorbidity in the general population.

Quantifying the extent of the joined prevalence of sarcopenia and cognitive impairment on adverse health outcomes is crucial to determine if participants with either disease should be screened for the respective other. Likewise, it is important to investigate if exploring a concurrent intervention—adhering to the approach of treating aging‐related diseases simultaneously [1]—would be reasonable as suggested based on previous cross‐sectional observations [5, 15, 16]. We therefore aimed to identify the prevalence of cognitive sarcopenia as well as the individual and joined association of sarcopenia and cognitive impairment with all‐cause and disease‐specific mortality during 12 years of follow‐up, as well as adverse outcomes including falls, hospitalization, disability, ADL disability, multimorbidity and requiring nursing care after 3 and 7 years of follow‐up in older adults from the general population.

## 2 Methods

### 2.1 Study Population

We analysed data from the population‐based cohort Cooperative Health Research in the Region Augsburg (KORA)‐Age (2008/9), which included participants born in 1943 or earlier who participated in any of the 4 preceding KORA studies Monitoring of Trends and Determinants in Cardiovascular Disease (MONICA) Augsburg S1, S2, S3 (1984–1995) or the KORA S4 study (1999–2001) [17]. The KORA‐Age study examination included 1079 participants aged 65–93 years. Those participants were invited to participate again in telephone interviews after 3 years (2012) and after 7 years (2016) of follow‐up.

Figure S1 illustrates the participant exclusions.

### 2.2 Sarcopenia and Cognitive Impairment

At baseline, probable and confirmed sarcopenia were defined according to the definition of the EWGSOP from 2018 [3] using grip strength and appendicular skeletal muscle mass (ASMM). Probable sarcopenia was defined as grip strength < 27 kg for men and < 16 kg for women. Confirmed sarcopenia was identified if probable sarcopenia was present and ASMM was < 20 kg for men and < 15 kg for women. Grip strength was measured three times performed with the dominant hand using a Jamar dynamometer (SAEHAN Corporation, Masan, Korea) in standing position with the elbow approximately 90° flexed. The maximum value of the three measurements was used for analyses [18]. ASMM was calculated from bioelectrical impedance analysis (BIA) measurements with the BIA 2000‐S (DATA‐INPUT GmbH, Frankfurt, Germany) using the equation by Sergi et al. [19] as recommended by the EWGSOP [3]. The BIA measurement was not performed if participants had a pacemaker and/or an amputation of the foot, leg, hand and/or arm. The measurement was performed in case of edema, endoprosthesis and/or metallic implants. The participants were asked to empty their bladder right before the measurement and to remove metallic objects such as keys and jewellery. Participants were not instructed to fast before the measurement.

Cognitive impairment was derived from the modified telephone interview for cognitive status (TICS‐m) or alternatively from a proxy‐interview with relatives or caregivers when participants had severe physical/mental impairment unable to conduct the interview themselves. If participants answered unreasonable/implausible, could not concentrate on the questions or did not understand the questions repeatedly, the proxy‐interview was implemented. The proxy‐interview included the ad8 informant interview with 10 questions regarding reduced memory or judgement ability of the participants comprising the answer “Yes” (1 point) or “No” (0 points) summed up to a single score. A sum of the score ≥ 2 defined cognitive impairment. Informants had the option to answer ‘I don't know’. A maximum of one unknown answer was allowed; otherwise, the score was set to missing. The validity and reliability of the ad8 has been demonstrated before [20]. The TICS‐m encompasses 48 items covering the domains orientation, memory, attention/calculation and language. This is an adjusted version of the TICS additionally including immediate and verbal recall [21]. The TICS‐m was corrected for education and categorized into three groups (18–27: impaired cognitive status, 28–31: mild impaired cognitive status, 32–52: good cognitive status) [22]. The implementation and feasibility of the TICS‐m score in KORA‐Age has been described in detail elsewhere [21]. We combined the participants with TICS‐m‐based mild and impaired cognitive status into one group. Since a proxy‐interview was implemented for the participants with missing TICS‐m due to severe physical/mental impairment, we combined the participants with available TICS‐m or with available proxy‐interview into one binary variable (no cognitive impairment versus cognitive impairment) to include the participants with severe impairments as well.

Cognitive probable sarcopenia was defined as having both probable sarcopenia and cognitive impairment; cognitive confirmed sarcopenia as having both confirmed sarcopenia and cognitive impairment. To allow the comparison of the isolated diseases with the co‐existence in the regression analyses, the reference group for all three risk groups (isolated probable sarcopenia, isolated cognitive impairment and cognitive probable sarcopenia) included only individuals with neither probable sarcopenia nor cognitive impairment (*N* = 709), respectively: (i) Isolated probable sarcopenia was defined as only having probable sarcopenia (*N* = 87) versus the individuals with neither probable sarcopenia nor cognitive impairment (*N* = 709); (ii) isolated cognitive impairment was defined as only having cognitive impairment (*N* = 174) versus the individuals with neither probable sarcopenia nor cognitive impairment (*N* = 709); (iii) cognitive probable sarcopenia was defined as having both probable sarcopenia and cognitive impairment (*N* = 85) versus the individuals with neither probable sarcopenia nor cognitive impairment (*N* = 709).

### 2.3 Sarcopenia‐Related Parameters

Muscle quality was calculated as grip strength in kg divided by ASMM in kg. Gait speed (m/s) was measured with the GAITRite system (CIR Systems, Havertown, PA, USA). The time to complete the Timed Up and Go Test (TUG) was measured in seconds as the time a participant needed to stand up from a chair, walk three metres, turn, walk back to the chair and sit down.

### 2.4 Mortality

Mortality was assessed between baseline and the end of the follow‐up on Dec. 31, 2021 using death certificates from local health authorities and the vital status from population registries. Cause‐specific mortality (cardiovascular disease [CVD], coronary heart disease [CHD]) was identified based on International Classification of Diseases (ICD)‐10 codes I00‐I99 and R96 for CVD mortality and I21‐I25 and R96 for CHD mortality. The follow‐up time was calculated from baseline examination until the date of death for deceased and for living persons until Dec. 31, 2021 or in rare cases until the date of last contact.

### 2.5 Adverse Health Outcomes

During telephone interviews in 2012 and 2016, participants were asked whether they had a fall, had been in the hospital for inpatient treatment and used nursing care insurance within 12 months before the telephone interview, respectively. A fall, hospital stay or nursing care was present when the respective event occurred at least once. The shorter period of 12 months was implemented to mitigate recall bias for longer periods. Disability and ADL disability were determined using the Health Assessment Questionnaire Disability Index (HAQ‐DI) encompassing 20 questions on 8 domains (eating, reaching, grasping, dressing, hygiene, getting up, walking and activity) on a 4‐point scale from 0 (*no difficulty*) to 3 (*unable to perform*). The items of each domain were combined using only the highest score for the respective domain. The HAQ‐DI is the mean of all 8 domains with a score of 0 meaning no disability up to a score of 3 representing severe disability. Having a disability was defined as HAQ‐DI > 0 [23]. ADL disability score was calculated by using the highest value [4‐point scale from 0 (*no difficulty*) to 3 (*unable to perform*)] of the three items (a) being able to run errands or do shopping yourself, (b) getting in and out of the car yourself and (c) to take care of the household yourself. ADL disability was defined if the score was 3. Multimorbidity was characterized as two or more diseases out of 13 chronic conditions (not including sarcopenia and cognitive impairment, dementia or similar) collected from a telephone interview and a self‐administered questionnaire [24]. The follow‐up time for adverse health outcomes comprises the time from baseline examination until 2012 and 2016, respectively. The loss to follow‐up for the outcomes and the number of events are reported in Table S1.

### 2.6 Covariables

Baseline covariables included age (years) at reference date (Dec. 31, 2008), sex (women/men), years of education (> 10 years/≤ 10 years), physical activity (active/inactive), nutrition, sleep duration (h/day), alcohol consumption (no consumption: 0 g/day / moderate: men 0.1–39.9 g/day, women 0.1–19.9 g/day / high: men ≥ 40 g/day, women ≥ 20 g/day), albumin (g/dL), arthritis (no/yes), neurological disease (no/yes), lung disease (no/yes) and polypharmacy (< 5 medications/≥ 5 medications). The nutrition score comprised the German version of the SCREEN II (Seniors in the community: risk evaluation for eating and nutrition, Version II). Physical activity was dichotomized as physically active/inactive based on questions regarding winter and summer leisure activity and exercise. Self‐reported sleep duration included how many hours participants usually sleep daily including midday naps. Serum albumin was measured using a modified bromocresol purple (BCP) dyebinding method with the Dimension Flex reagent cartridge ALB (Siemens Healthcare Diagnostics Inc.). Participants further self‐reported lung disease (such as asthma, emphysema, COPD), arthritis (such as arthritis, rheumatic disease) and neurological disease (such as multiple sclerosis, Parkinson's disease, epilepsy, but excluding stroke). Polypharmacy was defined as intake of ≥ 5 regular and prescribed drugs, excluding supplements, herbal and homeopathic products. Participants with depression were not excluded as there was no association with sarcopenia and cognitive impairment in this study.

### 2.7 Statistical Analysis

Venn diagrams illustrated the overlap of probable/confirmed sarcopenia with cognitive impairment at baseline. Logistic regression models assessed cross‐sectional associations of the sarcopenia‐related parameters with cognitive impairment and longitudinal associations of (isolated) probable sarcopenia, (isolated) cognitive impairment and cognitive probable sarcopenia with the six adverse health outcomes at follow‐up. The associations of these five probable sarcopenia/cognitive impairment definitions with all‐cause, CVD and CHD mortality were assessed using Cox proportional hazards regression models. The proportional hazards assumption was confirmed for all Cox regression models using scaled Schoenfeld residuals. All regression analyses were adjusted in three models. Software tools are described in the Supporting Information.

## 3 Results

Compared to the reference group (individuals without probable sarcopenia and without cognitive impairment), those with cognitive probable sarcopenia were older, less physically active, slept on average almost 2 h more per day and were more likely to have arthritis and/or a neurological disease (Table 1).

**TABLE 1:** Baseline characteristics of the study population.

Characteristic	All	Cognitive probable sarcopenia		
	*N* = 1055	Cases (*N* = 85)	Reference group^i^ (*N* = 709)	*p*
Sex, male [*N* (%)]^a^	524 (49.7)	47 (55.3)	337 (47.5)	0.216
Age, years [mean (SD)]^a^	76 (6.6)	82.4 (5.9)	74.3 (6.2)	**< 0.001**
Education, ≤ 10 years [*N* (%)]^a^	677 (64.2)	61 (71.8)	451 (63.6)	0.172
Physical activity, inactive [*N* (%)]^a^	497 (47.1)	67 (78.8)	291 (41.0)	**< 0.001**
Nutrition score [mean (SD)]^b^	38.1 (5.4)	36.4 (7.2)	38.7 (4.9)	**< 0.001**
Sleep, h/day [mean (SD)]^c^	7.9 (1.8)	9.4 (3.2)	7.6 (1.3)	**< 0.001**
Alcohol consumption [*N* (%)]^d^				**< 0.001**
No consumption	382 (36.4)	43 (54.4)	243 (34.3)	
Moderate	528 (50.3)	34 (43.0)	360 (50.8)	
High	139 (13.3)	2 (2.5)	106 (15.0)	
Albumin, g/dL [mean (SD)]^e^	3.8 (0.3)	3.6 (0.4)	3.8 (0.3)	**< 0.001**
Arthritis [*N* (%)]^a^	156 (14.8)	25 (29.4)	84 (11.8)	**< 0.001**
Neurological disease [*N* (%)]^a^	44 (4.2)	10 (11.8)	19 (2.7)	**< 0.001**
Lung disease [*N* (%)]^a^	114 (10.8)	15 (17.6)	67 (9.4)	**0.031**
Polypharmacy [*N* (%)]^a^	358 (33.9)	45 (52.9)	212 (29.9)	**< 0.001**
ASMM, kg [mean (SD)]^f^	19.4 (4.2)	18.4 (4.0)	19.5 (4.1)	**0.047**
Grip strength, kg [mean (SD)]^a^	27.7 (10.2)	15.6 (6.8)	30.0 (9.3)	**< 0.001**
TUG time, s [mean (SD)]^g^	10.8 (3.6)	14.5 (5.6)	10.1 (2.7)	**< 0.001**
Gait speed, m/s [mean (SD)]^h^	1.1 (0.2)	0.9 (0.2)	1.1 (0.2)	**< 0.001**
Muscle quality, kg/kg [mean (SD)]^f^	1.4 (0.4)	1.0 (0.3)	1.5 (0.3)	**< 0.001**

*Note: p*‐values for group differences were calculated using *T*‐test for continuous variables and chi‐squared test for categorical variables. Bold font for *p*‐values indicates significance (*p* ≤ 0.05). Cognitive probable sarcopenia was defined as having both probable sarcopenia and cognitive impairment.

Abbreviations: ASMM: appendicular skeletal muscle mass; TUG: Timed Up and Go Test.

^a^ Number of participants with available data: 1055.

^b^ Number of participants with available data: 1044.

^c^ Number of participants with available data: 1051.

^d^ Number of participants with available data: 1049.

^e^ Number of participants with available data: 1036.

^f^ Number of participants with available data: 986.

^g^ Number of participants with available data: 949.

^h^ Number of participants with available data: 935.

^i^ The reference group refers to those individuals without probable sarcopenia and without cognitive impairment.

### 3.1 Prevalence of Cognitive Sarcopenia

**FIGURE 1:**
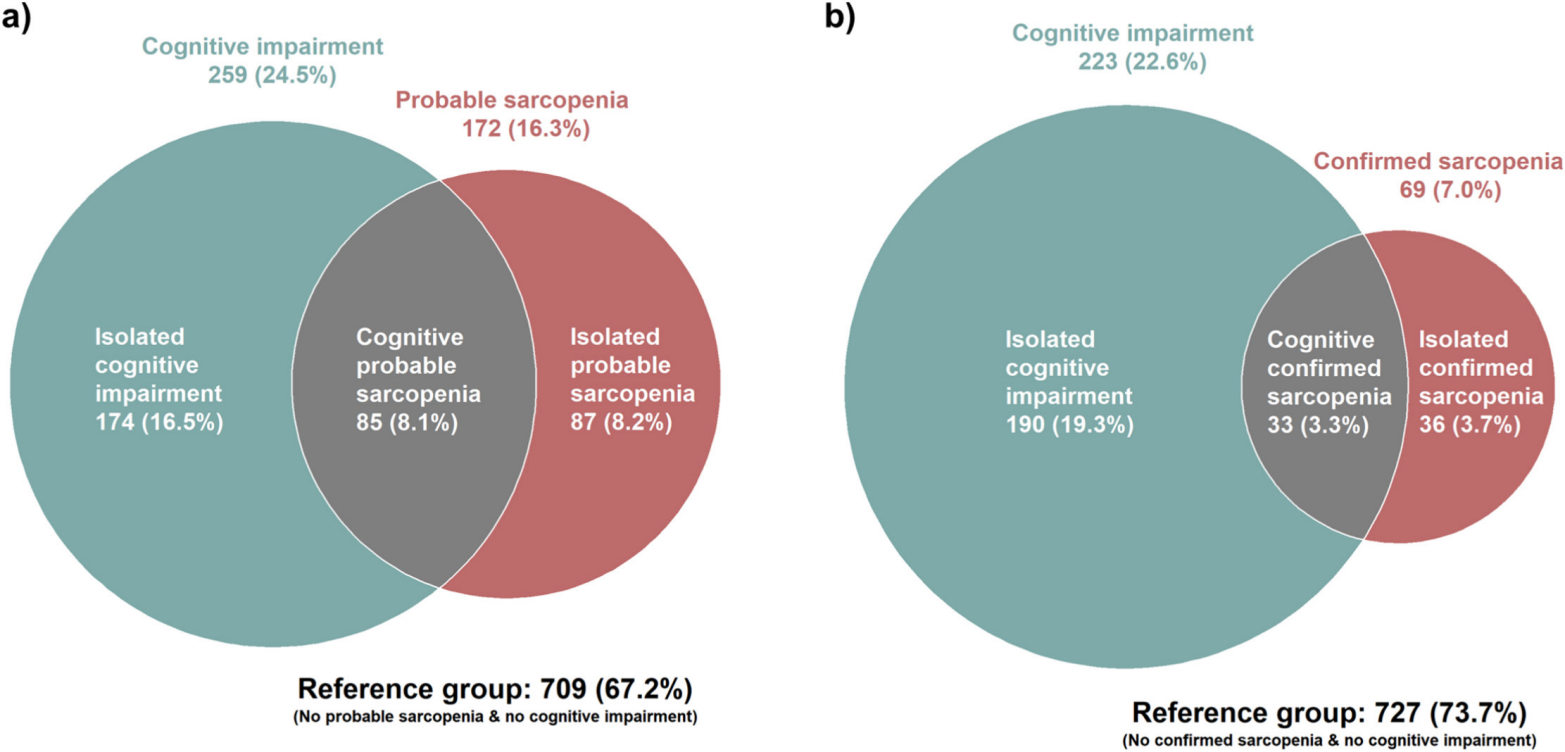
Venn diagram illustrating the overlap of cognitive impairment with (a) probable sarcopenia and (b) confirmed sarcopenia. Cognitive probable sarcopenia was defined as having both probable sarcopenia and cognitive impairment. Cognitive confirmed sarcopenia was defined as having both confirmed sarcopenia and cognitive impairment. 1055 participants were included for Venn diagram (a) and 986 participants for Venn diagram (b). For Venn diagram (b), 69 participants with missing values for appendicular skeletal muscle mass (ASMM) were excluded as ASMM is required to define confirmed sarcopenia.

A total of 8.1% of the baseline study population had cognitive probable sarcopenia, while only 3.3% had cognitive confirmed sarcopenia (Figure 1). Out of the participants with probable sarcopenia (*N* = 172), 49.4% (*N* = 85) had cognitive impairment, while out of the participants without probable sarcopenia (*N* = 883), only 19.7% (*N* = 174) had cognitive impairment.

### 3.2 Association of Sarcopenia‐Related Parameters With Cognitive Impairment

At baseline, muscle mass was the only sarcopenia parameter that was not associated with cognitive impairment, whereas grip strength, gait speed, TUG time (Models 1–3) and muscle quality (Models 1–2) were associated with cognitive impairment (Table 2). Because of the small overlap between confirmed sarcopenia and cognitive impairment (3.3%) (Figure 1) and the lacking association of muscle mass (component only of confirmed sarcopenia) with cognitive impairment (Table 2), following analyses with the longitudinal outcomes were not performed for cognitive confirmed sarcopenia.

**TABLE 2:** Logistic regression of the association between (z‐standardized) sarcopenia‐related parameters and cognitive impairment.

	Cognitive impairment	
	OR (95% CI)	*p*
ASMM ( *N* = 973)		
Model 1	0.93 (0.71, 1.21)	0.602
Model 2	0.95 (0.73, 1.25)	0.727
Model 3	0.92 (0.70, 1.20)	0.531
Grip strength (*N* = 1025)		
Model 1	0.64 (0.50, 0.82)	**< 0.001**
Model 2	0.69 (0.53, 0.88)	**0.003**
Model 3	0.73 (0.57, 0.94)	**0.017**
TUG time ( *N* = 946)		
Model 1	1.63 (1.38, 1.94)	**< 0.001**
Model 2	1.59 (1.35, 1.90)	**< 0.001**
Model 3	1.51 (1.27, 1.82)	**< 0.001**
Gait speed ( *N* = 933)		
Model 1	0.62 (0.51, 0.74)	**< 0.001**
Model 2	0.62 (0.51, 0.76)	**< 0.001**
Model 3	0.66 (0.54, 0.80)	**< 0.001**
Muscle quality (*N* = 973)		
Model 1	0.80 (0.66, 0.95)	**0.013**
Model 2	0.81 (0.67, 0.97)	**0.025**
Model 3	0.86 (0.71, 1.03)	0.106

*Note:* All sarcopenia‐related parameters were z‐standardized and are reported per standard deviation to allow the comparison of their effect estimates. Model adjustment: Model 1: Age, sex. Model 2: Model 1 + education, physical activity, nutrition, sleep duration, alcohol consumption. Model 3: Model 2 + albumin, arthritis, neurological disease, lung disease, polypharmacy. Bold font for *p*‐values indicates significance (*p* ≤ 0.05).

Abbreviations: ASMM: appendicular skeletal muscle mass; CI: confidence interval; OR: odds ratio; TUG: Timed Up and Go Test.

### 3.3 Association of Cognitive Probable Sarcopenia With Mortality and Adverse Outcomes

The total median follow‐up time was 11.5 years (9871 person‐years). Out of the 1055 participants from the total study population, 553 (52.4%) died (3782 person‐years). Of those, 110 (10.4%) died from CHD (702 person‐years) and 262 (24.8%) from CVD (1769 person‐years), while the information on cause of death was not available for 10 participants. Of those with isolated probable sarcopenia, 59 (67.8%) died (691 person‐years); of those with isolated cognitive impairment, 122 (70.1%) died (1489 person‐years); of those with cognitive probable sarcopenia, 78 (91.8%) died (450 person‐years); and in the reference group, 294 (41.5%) died (7238 person‐years).

While having cognitive probable sarcopenia at baseline increased the risk of all‐cause, CVD and CHD mortality independent of all covariables (Model 3), isolated cognitive impairment was only associated with all‐cause mortality after full adjustment (Table 3).

**TABLE 3:** Association of isolated probable sarcopenia, isolated cognitive impairment and cognitive probable sarcopenia with mortality during 12 years of follow‐up (Cox regression models).

	Isolated probable sarcopenia vs. reference group^a^		Isolated cognitive impairment vs. reference group^a^		Cognitive probable sarcopenia vs. reference group^a^	
	HR (95% CI)	*p*	HR (95% CI)	*p*	HR (95% CI)	*p*
All‐cause mortality						
Model 1	1.38 (1.03, 1.86)	**0.032**	1.54 (1.24, 1.91)	**< 0.001**	2.12 (1.55, 2.90)	**< 0.001**
Model 2	1.30 (0.96, 1.76)	0.091	1.49 (1.20, 1.86)	**< 0.001**	1.97 (1.42, 2.74)	**< 0.001**
Model 3	1.31 (0.96, 1.80)	0.088	1.39 (1.11, 1.73)	**0.004**	1.95 (1.41, 2.70)	**< 0.001**
CVD mortality						
Model 1	1.18 (0.76, 1.81)	0.462	1.40 (1.02, 1.92)	**0.040**	1.69 (1.07, 2.67)	**0.025**
Model 2	1.14 (0.73, 1.76)	0.569	1.34 (0.97, 1.86)	0.075	1.63 (1.01, 2.64)	**0.044**
Model 3	1.18 (0.75, 1.86)	0.485	1.25 (0.90, 1.75)	0.177	1.64 (1.02, 2.64)	**0.042**
CHD mortality						
Model 1	1.62 (0.86, 3.05)	0.135	1.48 (0.90, 2.43)	0.122	2.15 (1.09, 4.27)	**0.028**
Model 2	1.63 (0.86, 3.10)	0.138	1.48 (0.89, 2.46)	0.127	2.27 (1.12, 4.62)	**0.024**
Model 3	1.84 (0.94, 3.57)	0.073	1.40 (0.84, 2.34)	0.194	2.10 (1.03, 4.27)	**0.040**

*Note:* Model adjustment: Model 1: Age, sex. Model 2: Model 1 + education, physical activity, nutrition, sleep duration, alcohol consumption. Model 3: Model 2 + albumin, arthritis, neurological disease, lung disease, polypharmacy. Bold font for *p*‐values indicates significance (*p* ≤ 0.05). Cognitive probable sarcopenia was defined as having both probable sarcopenia and cognitive impairment.

Abbreviations: CHD: coronary heart disease; CI: confidence interval; CVD: cardiovascular disease; HR: hazard ratio.

^a^ The reference group refers to those individuals without probable sarcopenia and without cognitive impairment (see Figure 1).

**FIGURE 2:**
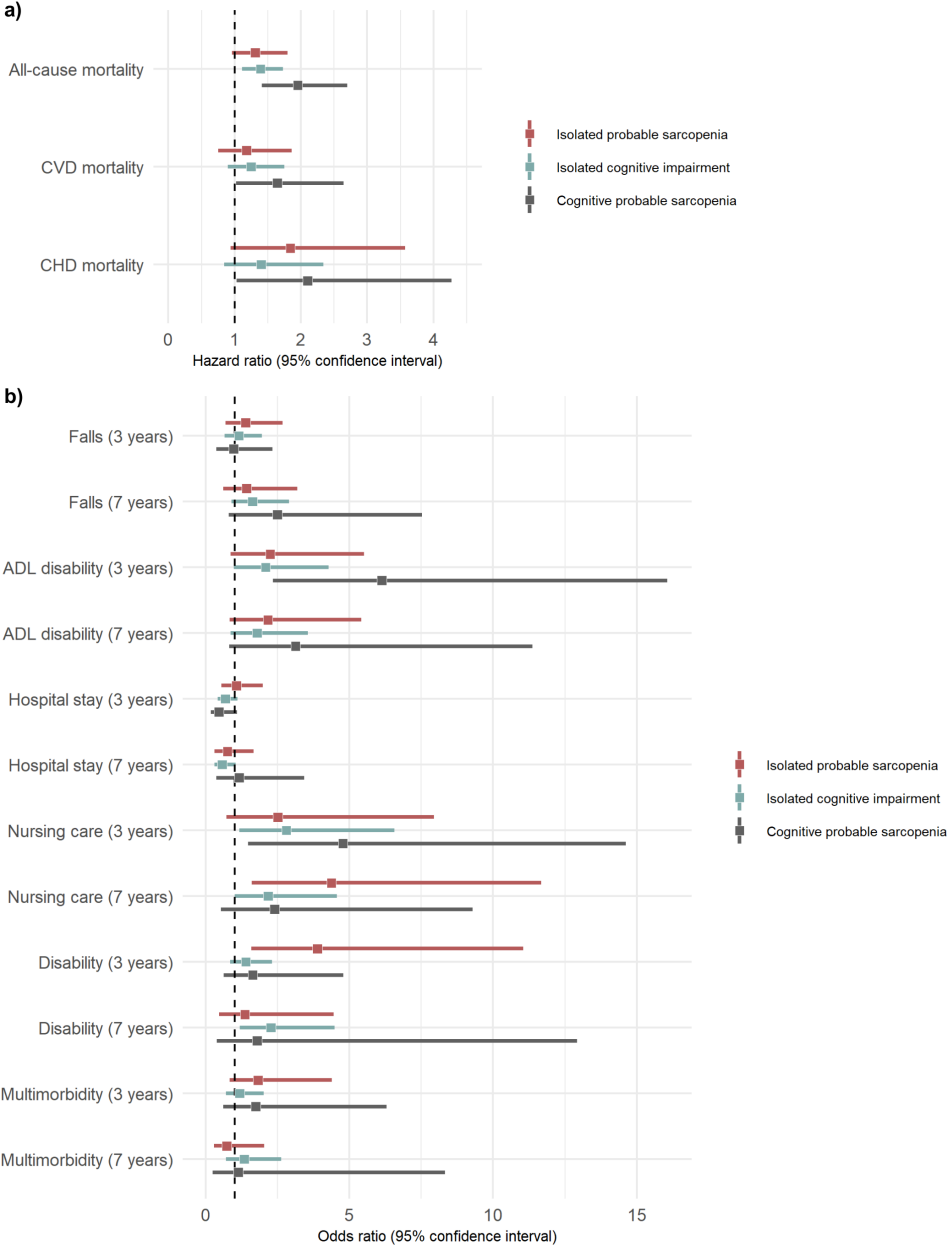
Forest plots of the association of isolated probable sarcopenia, isolated cognitive impairment and cognitive probable sarcopenia with (a) mortality and (b) adverse outcomes in Model 3. ADL: activities of daily living; CHD: coronary heart disease; CVD: cardiovascular disease. All models were adjusted for age, sex, education, physical activity, nutrition, sleep duration, alcohol consumption, albumin, arthritis, neurological disease, lung disease and polypharmacy (Model 3). Cognitive probable sarcopenia was defined as having both probable sarcopenia and cognitive impairment.

Compared to individuals with the isolated diseases, participants with cognitive probable sarcopenia had an increased risk of premature all‐cause, CVD and CHD mortality (Table 3 and Figure 2a) as well as ADL disability and requiring nursing care after 3 years (Table 4 and Figure 2b). Participants with isolated probable sarcopenia had increased odds of disability after 3 years, whereas participants with isolated cognitive impairment had increased odds of disability after 7 years in Model 3. Isolated cognitive impairment showed positive associations with nursing care after 3 and 7 years, while isolated probable sarcopenia was positively associated with nursing care only after 7 years. Falls, hospital stay and multimorbidity did not show any associations with all three risk groups.

**TABLE 4:** Association of isolated probable sarcopenia, isolated cognitive impairment and cognitive probable sarcopenia with adverse outcomes at 3 and 7 years of follow‐up (logistic regression models).

	Isolated probable sarcopenia vs. reference group^a^		Isolated cognitive impairment vs. reference group^a^		Cognitive probable sarcopenia vs. reference group^a^	
	OR (95% CI)	*p*	OR (95% CI)	*p*	OR (95% CI)	*p*
Falls (3 years)						
Model 1	1.45 (0.75, 2.71)	0.255	1.29 (0.75, 2.14)	0.344	0.89 (0.35, 2.05)	0.789
Model 2	1.48 (0.75, 2.81)	0.241	1.24 (0.72, 2.08)	0.429	1.01 (0.39, 2.39)	0.987
Model 3	1.38 (0.69, 2.68)	0.346	1.15 (0.66, 1.95)	0.619	0.97 (0.37, 2.32)	0.941
Falls (7 years)						
Model 1	1.53 (0.68, 3.23)	0.284	1.66 (0.94, 2.90)	0.077	2.88 (0.94, 8.47)	0.055
Model 2	1.53 (0.67, 3.30)	0.295	1.64 (0.91, 2.90)	0.090	2.81 (0.91, 8.32)	0.062
Model 3	1.42 (0.60, 3.18)	0.412	1.62 (0.89, 2.89)	0.104	2.49 (0.80, 7.53)	0.105
ADL disability (3 years)						
Model 1	2.09 (0.87, 4.73)	0.086	2.47 (1.21, 4.91)	**0.011**	5.99 (2.46, 14.39)	**< 0.001**
Model 2	2.10 (0.85, 4.93)	0.098	2.26 (1.08, 4.59)	**0.026**	5.44 (2.13, 13.66)	**< 0.001**
Model 3	2.24 (0.87, 5.51)	0.086	2.08 (0.98, 4.28)	0.051	6.12 (2.33, 16.06)	**< 0.001**
ADL disability (7 years)						
Model 1	2.04 (0.84, 4.78)	0.105	1.88 (0.96, 3.63)	0.062	3.48 (0.97, 11.66)	**0.047**
Model 2	2.14 (0.85, 5.22)	0.100	1.78 (0.87, 3.55)	0.109	4.30 (1.14, 14.98)	**0.024**
Model 3	2.16 (0.83, 5.41)	0.105	1.78 (0.87, 3.56)	0.109	3.12 (0.81, 11.37)	0.087
Hospital stay (3 years)						
Model 1	1.08 (0.57, 1.95)	0.813	0.84 (0.52, 1.33)	0.477	0.56 (0.22, 1.29)	0.199
Model 2	1.06 (0.56, 1.94)	0.862	0.78 (0.48, 1.24)	0.310	0.51 (0.19, 1.21)	0.150
Model 3	1.06 (0.54, 1.99)	0.865	0.68 (0.41, 1.11)	0.131	0.46 (0.17, 1.09)	0.096
Hospital stay (7 years)						
Model 1	0.88 (0.38, 1.87)	0.751	0.60 (0.32, 1.08)	0.098	1.37 (0.44, 3.89)	0.566
Model 2	0.79 (0.34, 1.70)	0.558	0.58 (0.31, 1.04)	0.080	1.28 (0.40, 3.70)	0.658
Model 3	0.75 (0.31, 1.67)	0.495	0.57 (0.30, 1.04)	0.076	1.16 (0.37, 3.43)	0.786
Nursing care (3 years)						
Model 1	2.46 (0.79, 6.84)	0.097	3.96 (1.76, 8.83)	**0.001**	6.86 (2.32, 19.43)	**< 0.001**
Model 2	2.16 (0.67, 6.23)	0.172	3.48 (1.50, 7.95)	**0.003**	5.07 (1.57, 15.28)	**0.005**
Model 3	2.51 (0.72, 7.95)	0.127	2.80 (1.17, 6.57)	**0.018**	4.77 (1.47, 14.63)	**0.007**
Nursing care (7 years)						
Model 1	3.85 (1.55, 9.25)	**0.003**	2.34 (1.13, 4.75)	**0.020**	2.16 (0.49, 8.01)	0.269
Model 2	3.94 (1.53, 9.88)	**0.004**	2.16 (1.01, 4.51)	**0.044**	2.81 (0.61, 10.69)	0.150
Model 3	4.37 (1.60, 11.68)	**0.003**	2.17 (1.01, 4.56)	**0.043**	2.39 (0.52, 9.29)	0.227
Disability (3 years)						
Model 1	4.56 (1.98, 12.47)	**0.001**	1.56 (0.99, 2.50)	0.058	2.21 (0.91, 6.23)	0.101
Model 2	4.05 (1.71, 11.24)	**0.003**	1.55 (0.96, 2.52)	0.074	2.03 (0.81, 5.85)	0.156
Model 3	3.88 (1.59, 11.05)	**0.005**	1.39 (0.85, 2.31)	0.193	1.63 (0.63, 4.79)	0.336
Disability (7 years)						
Model 1	1.96 (0.76, 5.82)	0.186	2.17 (1.19, 4.10)	**0.014**	3.29 (0.80, 22.41)	0.141
Model 2	1.66 (0.61, 5.10)	0.343	2.31 (1.24, 4.46)	**0.010**	2.68 (0.66, 18.09)	0.220
Model 3	1.36 (0.47, 4.45)	0.585	2.27 (1.19, 4.48)	**0.015**	1.78 (0.39, 12.93)	0.501
Multimorbidity (3 years)						
Model 1	2.07 (0.99, 4.88)	0.069	1.30 (0.81, 2.15)	0.284	2.13 (0.80, 7.41)	0.171
Model 2	1.85 (0.88, 4.38)	0.130	1.31 (0.81, 2.18)	0.281	2.02 (0.74, 7.08)	0.210
Model 3	1.81 (0.83, 4.39)	0.159	1.18 (0.71, 2.01)	0.531	1.74 (0.61, 6.29)	0.339
Multimorbidity (7 years)						
Model 1	1.02 (0.44, 2.67)	0.959	1.35 (0.74, 2.59)	0.348	1.72 (0.45, 11.29)	0.490
Model 2	0.89 (0.38, 2.36)	0.809	1.31 (0.71, 2.54)	0.407	1.77 (0.45, 11.86)	0.470
Model 3	0.73 (0.29, 2.03)	0.524	1.34 (0.71, 2.63)	0.384	1.13 (0.24, 8.32)	0.886

*Note:* Model adjustment: Model 1: Age, sex. Model 2: Model 1 + education, physical activity, nutrition, sleep duration, alcohol consumption. Model 3: Model 2 + albumin, arthritis, neurological disease, lung disease, polypharmacy. Bold font for *p*‐values indicates significance (*p* ≤ 0.05). Cognitive probable sarcopenia was defined as having both probable sarcopenia and cognitive impairment.

Abbreviations: ADL: activities of daily living; CI: confidence interval; OR: odds ratio.

^a^ The reference group refers to those individuals without probable sarcopenia and without cognitive impairment (see Figure 1).

For comparability to other studies, which did not focus on cognitive probable sarcopenia, and did therefore not investigate the isolated definitions, Tables S2 and S3 list the associations of probable sarcopenia versus all remaining participants and cognitive impairment versus all remaining participants. Interaction between probable sarcopenia and cognitive impairment was indicated due to concurrent positive associations of both exposures with all‐cause mortality (Models 1–3), CHD mortality (Model 1), ADL disability after 3 years (Models 1–3) and nursing care after 3 years (Model 3).

## 4 Discussion

Almost 50% of older adults with probable sarcopenia had cognitive impairment. Muscle mass was not associated with cognitive impairment, whereas having weaker grip strength, slower gait speed and longer TUG time were associated with increased odds of having cognitive impairment. A total of 8.1% of the baseline population had cognitive probable sarcopenia, while only 3.3% had cognitive confirmed sarcopenia. Participants with cognitive probable sarcopenia had an increased risk of all‐cause, CVD and CHD mortality after 12 years, and ADL disability and requiring nursing care after 3 years. Participants with only isolated probable sarcopenia or isolated cognitive impairment had lower or no significant risk for those outcomes.

### 4.1 Almost 50% of Older Adults With Probable Sarcopenia Had Cognitive Impairment

In the present study population of older adults from the general population, 49.4% of individuals with probable sarcopenia had cognitive impairment, while among participants without probable sarcopenia only 19.7% had cognitive impairment. A similar trend was reported in a meta‐analysis from 2019 in which cognitive impairment was present in 40% of participants with sarcopenia but only in 25.3% of participants without sarcopenia [8]. Another meta‐analysis observed that 20.5% of participants with sarcopenia had mild cognitive impairment (MCI), while only 9.1% of participants with MCI had sarcopenia based on diverse data including community and hospital participants as well as different ethnicities and definitions of sarcopenia [7]. This observation supports our secondary findings that the prevalence of cognitive impairment in individuals with probable or confirmed sarcopenia is higher than vice versa. Yet, a more recent meta‐analysis from 2024 highlighted the wide range of sarcopenia prevalence in older adults with MCI from 2.8% to 73.9% and similarly of the MCI prevalence in older adults with sarcopenia ranging between 10.5% and 71.0% [9], emphasizing the large variation of the prevalence in different study populations and study designs such as the definition of sarcopenia and MCI.

### 4.2 8.1% of Older Adults Had Cognitive Probable Sarcopenia

A total of 8.1% of this study population had cognitive probable sarcopenia, while only 3.3% had cognitive confirmed sarcopenia. The above‐mentioned meta‐analyses did not report the pooled prevalence of cognitive sarcopenia in the whole study population. However, in individual studies for instance in patients discharged from acute care hospitals in Italy (mean age 80 years) approximately 9.8% had cognitive confirmed sarcopenia (with sarcopenia based on EWGSOP 2018) [12], while in American adults aged 50+ only 4.3% had cognitive probable sarcopenia (with low grip strength defined by American‐specific cut points) [14]. Even though the definitions of sarcopenia vary, these results seem to align with our study since it can be expected that the prevalence of cognitive sarcopenia is higher in former hospital patients [12] and lower in younger adults [14].

### 4.3 Muscle Function but Not Muscle Mass Was Associated With Cognitive Impairment

The observation that muscle mass was not, but all muscle function parameters were associated with cognitive impairment confirms the consensus of previous findings. A study of older US‐American adults concluded that muscle function rather than muscle mass may impact the association between sarcopenia and incident cognitive impairment, since muscle mass was not associated with this outcome when adjusting for muscle function [25]. In a Singaporean study that assessed individual sarcopenia components, appendicular lean mass index was not, but low hand grip strength was associated with cognitive impairment [26]. In line with these results, another cross‐sectional study reported that cognitive impairment was associated with muscle function, but not muscle mass in Turkish adults [27]. Similarly, in middle‐aged and older US‐American adults, low grip strength was, but low muscle mass was not associated with a measure of early cognitive changes [16]. The pathophysiology of cognitive sarcopenia is still unclear; however, brain atrophy in areas such as the parietal lobe has been discussed [11], which might promote lower muscle control rather than lower muscle mass. Additionally, reduced physical activity and mobility due to cognitive impairment [11] might initially limit muscle movement rather than muscle mass.

Along with various other studies, our findings suggest that screening for cognitive impairment is reasonable in older adults with sarcopenia defined by muscle function, while the benefit of adding muscle mass should be investigated in larger sample sizes and therefore a higher number of cases.

### 4.4 Participants With Cognitive Probable Sarcopenia Had an Increased Risk of All‐Cause, CVD and CHD Mortality, ADL Disability and Requiring Nursing Care

Having cognitive probable sarcopenia increased the risk of premature all‐cause, CVD and CHD mortality. Individuals with isolated probable sarcopenia did not have an increased risk for mortality, while isolated cognitive impairment was only associated with all‐cause mortality after full covariable adjustment. Comparable findings were described in older patients discharged from acute care in Italy after 12 months of follow‐up [12], which demonstrated for both the EWGSOP sarcopenia definition from 2010 and 2018 that cognitive confirmed sarcopenia was associated with all‐cause mortality. Additionally, in older adults from the US population, cognitive confirmed sarcopenia (with sarcopenia defined by ASMM relative to body mass index) was associated with mortality related to cardiovascular and cerebrovascular diseases and Alzheimer's disease after a medium follow‐up of 48 months [13]. Beyond sarcopenia, an increased mortality risk was further reported for Swedish older adults with both cognitive impairment (without dementia) and a slow walking speed [28].

Cognitive probable sarcopenia was associated with ADL disability after 3 years, while isolated probable sarcopenia and isolated cognitive impairment were not associated with ADL disability. Only one other study reported directly comparable results and found that cognitive probable sarcopenia (with low grip strength defined by American‐specific cut points) increased the odds of ADL disability compared to any of the diseases individually in middle‐aged and older Americans [14]. Further related findings cover that grip strength, but not appendicular lean mass, was inversely associated with a higher risk of hospital‐associated ADL disability, with a stronger association in older US‐American adults with cognitive impairment [29]. Similarly, in a Japanese study, sarcopenia defined by both muscle mass and function was associated with ADL impairment in early‐stage Alzheimer's disease [30].

Cognitive probable sarcopenia was associated with requiring nursing care after 3 years, while after 7 years only the isolated definitions showed associations with nursing care. Interestingly, we found time‐varying results for the outcome of disability as participants with isolated probable sarcopenia had increased odds of disability after 3 years, whereas participants with isolated cognitive impairment had increased odds of disability after 7 years. Comparable results have only been reported in a Swedish study that observed an increased disability in participants having both cognitive impairment (without dementia) and a slow walking speed, which was also attenuated after longer follow‐up times over 12 years [28]. While it has been suggested that the reduction in cognition and physical parameters may be viewed as a common rather than a parallel way leading to disability [31], the present results only confirm this for ADL disability but not for disability in general.

Both (non‐isolated) probable sarcopenia and cognitive impairment were positively associated with all‐cause mortality, ADL disability and nursing care after 3 years (Model 3). Thus, the interaction between probable sarcopenia and cognitive impairment was present for those outcomes, since the concurrent effects of both exposures for the same outcome are sufficient to demonstrate interaction [32]. However, larger sample sizes are needed to show the significance of the interaction than of the overall effects [32].

The lacking association of cognitive probable sarcopenia with falls in this study was unexpected since in a cross‐sectional study in Italy, 91% of participants with cognitive probable sarcopenia showed balance deficits compared to 38% of participants without both diseases [33]. A recent meta‐analysis of four studies concluded that individuals with cognitive impairment that experienced falls have a higher risk of sarcopenia [34]. Further unexpected were the non‐significant associations between all three risk groups and hospital stays. This finding may be explained by assuming that those individuals are rather admitted to care facilities than the hospital since sarcopenia and cognitive impairment may not immediately lead to hospitalization. This also fits to the overall observation of this study that cognitive sarcopenia seems to be more strongly related to loss of independence (ADL disability and nursing care) and reduction in lifespan rather than to acute endpoints such as falls and hospital stays. Considering the aging population and the increasing demand for costly and long‐term resources such as nursing care, targeting cognitive sarcopenia as a joint concept could be considered to mitigate those strained resources.

In line with the conclusions of previous cross‐sectional studies [5, 15, 16], our longitudinal results manifest the suggestion to screen for cognitive impairment when probable sarcopenia is present and support the consensus to target diseases during aging simultaneously in respect to probable sarcopenia and cognitive impairment. Further emphasizing this approach, a recent meta‐analysis concluded that multidomain interventions in older persons covering both physical and cognitive training improved muscle strength and cognitive flexibility (which refers to the adaptation of cognitive processes due to changing circumstances) [35]. Yet, considering combined interventions, patients with cognitive impairment may require specific protocols to ensure compliance when implementing physical activity or nutrition interventions [36].

The baseline of this study was collected more than a decade ago. However, life expectancy has not considerably improved since baseline (2008/9) until 2024 in Germany [37]. According to a recent meta‐analysis, survival with dementia remained unchanged in community‐based studies over the past decades [38]. Data on effective treatment for sarcopenia is lacking, while low diagnostic rates in recent years [39] do not indicate widespread treatment. Due to the lack of considerate healthcare changes relevant to this data, we expect that this article's results are still relevant today.

### 4.5 Strengths and Limitations

Strengths of the study include the population‐based design and the implementation of a proxy interview with relatives/caregivers for participants that were unable to answer the interview questions themselves. This allowed us to include participants with severe cognitive impairment, who are usually excluded from other population‐based studies. In addition, the evaluation of two follow‐up time points allowed us to evaluate the adverse outcomes for a shorter and a longer follow‐up time. Another strength covers the inclusion of three functional sarcopenia parameters (grip strength, TUG time and gait speed), which enabled us to substantiate the conclusion that muscle function was robustly associated with cognitive impairment. Limitations include the loss to follow‐up, which could have influenced the findings of the adverse outcomes, especially regarding the differences observed between the 3‐ and 7‐year time periods. However, mortality status was available for the complete study sample. Another limitation is the sample size of the population with confirmed sarcopenia. Larger study populations are needed to assess the impact on cognitive impairment when muscle mass is added in the definition of sarcopenia and to assess the size of multiplicative interactions. The adverse outcomes such as falls were based on self‐report, which could have resulted in an underestimation of those events. Telephone‐based cognitive assessments do not include neurological and visuospatial examinations and could be influenced by reduced attention and hearing problems [21], whereas they are more appropriate for persons with visual deficits. The generalizability of the results may be limited to White Europeans.

## 5 Conclusion

Half of the participants with probable sarcopenia had cognitive impairment. Having cognitive probable sarcopenia compared to the isolated diseases was associated with an increased risk of all‐cause, CVD and CHD mortality after 12 years and ADL disability and requiring nursing care after 3 years. Cognitive sarcopenia seems to be more strongly related to a reduction in lifespan and loss of independence of older people rather than to acute outcomes including hospitalization and falls. Considering the increasing need of the aging population for care resources, our findings endorse the prevention approach to screen for cognitive impairment in older adults with probable sarcopenia and support prior suggestions for exploring intervention studies targeting both diseases simultaneously.

## Funding

The KORA study was initiated and financed by the Helmholtz Zentrum München—German Research Center for Environmental Health, which is funded by the German Federal Ministry of Education and Research (BMBF) and by the State of Bavaria. Data collection in the KORA study is done in cooperation with the University Hospital of Augsburg. The KORA‐Age project was financed by the German Federal Ministry of Education and Research (BMBF FKZ 01ET0713 and 01ET1003A) as part of the ‘Health in old age’ programme.

## Ethics Statement

The ethics committee of the Bavarian Medical Association approved the KORA study, which was performed in accordance with the ethical standards laid down in the 1964 Declaration of Helsinki and its later amendments. All participants gave their written informed consent. All authors of this manuscript comply with the guidelines of ethical authorship and publishing in the Journal of Cachexia, Sarcopenia and Muscle [40].

## Conflicts of Interest

The authors declare no conflicts of interest.

## Supporting information




**Figure S1:** Flow chart illustrating the exclusion of participants.
**Table S1:** Available sample size and number of events for mortality and adverse outcomes (*N* = 1025).
**Table S2:** Association of probable sarcopenia and cognitive impairment with mortality during 12 years of follow‐up (Cox regression models).
**Table S3:** Association of probable sarcopenia and cognitive impairment with adverse outcomes at 3 and 7 years of follow‐up (logistic regression models).

## Data Availability

The informed consent given by KORA study participants does not cover data posting in public databases. However, data are available upon request from KORA.PASST (https://helmholtz‐muenchen.managed‐otrs.com/external/) by means of a project agreement. Requests should be sent to kora.passt@helmholtz-munich.de and are subject to approval by the KORA Board.
