# Supplementary material for: Cognitive Sarcopenia: Prevalence and the Risk for Mortality and Healthy Aging in the KORA‐Age Study
Source: J Cachexia Sarcopenia Muscle. 2026 Jan 25;17(1):e70201. doi: 10.1002/jcsm.70201 (PMC12833496; doi:10.1002/jcsm.70201)
Supplement: Supplementary file 1 — Figure S1: Flow chart illustrating the exclusion of participants. Table S1: Available sample size and number of events for mortality and adverse outcomes (N = 1025). Table S2: Association of probable sarcopenia and cognitive impairment with mortality during 12 years of follow‐up (Cox regression models). Table S3: Association of probable sarcopenia and cognitive impairment with adverse outcomes at 3 and 7 years of follow‐up (logistic regression models). [file JCSM-17-e70201-s001.pdf]

Supporting Information

Cognitive sarcopenia: Prevalence and the risk for mortality and healthy aging in the KORA-Age study

Marie-Theres Huemer[1, 2], Barbara Thorand[1, 3], Eva Grill[3, 4], Lars Schwettmann[5, 6], Annette Peters[1, 3]

[1] Institute of Epidemiology, Helmholtz Zentrum München, German Research Center for Environmental Health (GmbH), Ingolstädter Landstraße 1, 85764, Neuherberg, Germany

[2] Department of Epidemiology, Harvard T.H. Chan School of Public Health, Boston, USA

[3] Institute for Medical Information Processing, Biometry and Epidemiology, Medical Faculty, Ludwig-Maximilians-Universität München, Marchioninistr. 15, 81377, Munich, Germany

[4] German Center for Vertigo and Balance Disorders, DSGZ, Faculty of Medicine, Ludwig-Maximilians Universität Munich (LMU), Germany

[5] Institute of Health Economics and Health Care Management, Helmholtz Zentrum München, German Research Center for Environmental Health (GmbH), Ingolstädter Landstraße 1, 85764, Neuherberg, Germany

[6] Department of Health Services Research, School of Medicine and Health Sciences, Carl von Ossietzky University of Oldenburg, Ammerländer Heerstr. 114-118, 26129 Oldenburg, Germany

Corresponding author:

Marie-Theres Huemer
Ingolstädter Landstr.1, 85764 Neuherberg, Germany
marietheres.huemer@helmholtz-munich.de

Table of contents

Fig. S1 Flow chart illustrating the exclusion of participants

Table S1 Available sample size and number of events for mortality and adverse outcomes (N = 1025)

Software tools

Table S2 Association of probable sarcopenia and cognitive impairment with mortality during twelve years of follow-up (Cox regression models)

Table S3 Association of probable sarcopenia and cognitive impairment with adverse outcomes at three and seven years of follow-up (logistic regression models)

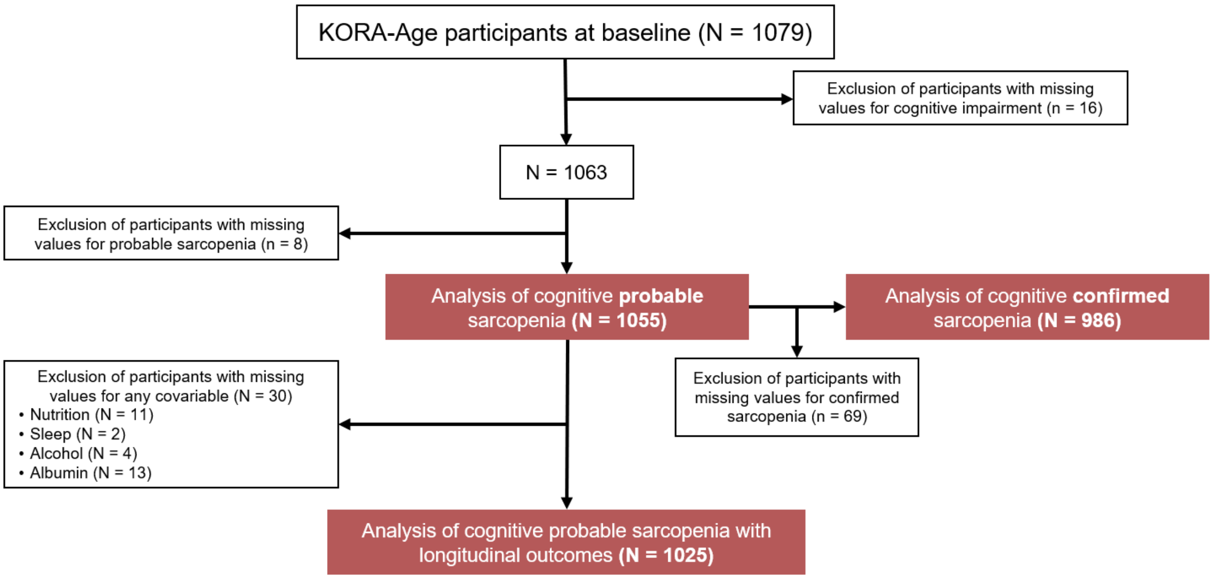

Fig. S1 Flow chart illustrating the exclusion of participants

Out of the 1079 baseline participants, 16 participants with missing values for cognitive impairment and 8 participants with missing values for probable sarcopenia were excluded. The remaining 1055 participants were included in the analysis of cognitive probable sarcopenia. For all analyses that included adjustment for covariables, we further excluded 30 participants with missing values for any covariable (N=1025). For the analysis of cognitive confirmed sarcopenia, we additionally excluded 69 participants out of the 1055 with missing values for appendicular skeletal muscle mass (ASMM), as ASMM is required to confirm sarcopenia (N=986).

Table S1 Available sample size and number of events for mortality and adverse outcomes (N = 1025)

Outcome	Available sample size (N)	Events [N (%)]
All-cause mortality (12 years)	1025	526 (51.3)
CVD mortality (12 years)	1015	247 (24.3)
CHD mortality (12 years)	1015	103 (10.1)
Falls (3 years)	800	142 (17.8)
Falls (7 years)	600	125 (20.8)
ADL disability (3 years)	791	70 (8.8)
ADL disability (7 years)	599	89 (14.9)
Hospital stay (3 years)	789	205 (26.0)
Hospital stay (7 years)	600	145 (24.2)
Nursing care (3 years)	790	42 (5.3)
Nursing care (7 years)	599	65 (10.9)
Disability (3 years)	791	495 (62.6)
Disability (7 years)	599	385 (64.3)
Multimorbidity (3 years)	790	575 (72.8)
Multimorbidity (7 years)	593	448 (75.5)

ADL: activities of daily living; CHD: coronary heart disease; CVD: cardiovascular disease

Software tools

All statistical analyses were performed using R, V 4.3.1 [1]. The R package “eulerr” was used to create the Venn diagrams [2], the R package “survival” [3] to calculate the Cox regression models.

Table S2 Association of probable sarcopenia and cognitive impairment with mortality during twelve years of follow-up (Cox regression models)

	**Probable sarcopenia** vs all remaining participants		**Cognitive impairment** vs all remaining participants	
	HR (95% CI)	p-value	HR (95% CI)	p-value
**All-cause mortality**				
Model 1	1.46 (1.17, 1.81)	**0.001**	1.60 (1.32, 1.93)	**<0.001**
Model 2	1.31 (1.04, 1.64)	**0.019**	1.54 (1.27, 1.86)	**<0.001**
Model 3	1.30 (1.03, 1.63)	**0.024**	1.46 (1.20, 1.77)	**<0.001**
**CVD mortality**				
Model 1	1.27 (0.92, 1.75)	0.145	1.46 (1.11, 1.93)	**0.007**
Model 2	1.13 (0.81, 1.58)	0.456	1.41 (1.06, 1.88)	**0.019**
Model 3	1.16 (0.83, 1.62)	0.380	1.33 (1.00, 1.78)	**0.049**
**CHD mortality**				
Model 1	1.68 (1.04, 2.70)	**0.034**	1.56 (1.02, 2.38)	**0.040**
Model 2	1.60 (0.98, 2.61)	0.060	1.55 (1.00, 2.39)	**0.050**
Model 3	1.72 (1.05, 2.81)	**0.030**	1.46 (0.94, 2.27)	0.090

CHD: coronary heart disease; CI: confidence interval; CVD: cardiovascular disease; HR: hazard ratio.

Model adjustment:
Model 1: Age, sex.
Model 2: Model 1 + education, physical activity, nutrition, sleep duration, alcohol consumption.
Model 3: Model 2 + albumin, arthritis, neurological disease, lung disease, polypharmacy.

Bold font for p-values indicates significance ($p \leq 0.05$).

Table S3 Association of probable sarcopenia and cognitive impairment with adverse outcomes at three and seven years of follow-up (logistic regression models)

	**Probable sarcopenia** vs all remaining participants		**Cognitive impairment** vs all remaining participants	
	OR (95% CI)	p-value	OR (95% CI)	p-value
**Falls (3 years)**				
Model 1	1.19 (0.69, 1.99)	0.528	1.13 (0.70, 1.79)	0.604
Model 2	1.21 (0.69, 2.05)	0.495	1.12 (0.69, 1.78)	0.652
Model 3	1.18 (0.67, 2.02)	0.564	1.06 (0.65, 1.70)	0.818
**Falls (7 years)**				
Model 1	1.63 (0.85, 3.04)	0.128	1.69 (1.00, 2.79)	**0.045**
Model 2	1.57 (0.81, 2.96)	0.167	1.69 (1.00, 2.82)	**0.046**
Model 3	1.42 (0.72, 2.71)	0.297	1.65 (0.97, 2.78)	0.060
**ADL disability (3 years)**				
Model 1	2.78 (1.50, 5.04)	**0.001**	2.92 (1.65, 5.18)	**<0.001**
Model 2	2.53 (1.34, 4.68)	**0.004**	2.59 (1.43, 4.67)	**0.002**
Model 3	2.84 (1.48, 5.37)	**0.001**	2.41 (1.32, 4.38)	**0.004**
**ADL disability (7 years)**				
Model 1	2.02 (0.97, 4.09)	0.054	1.85 (1.00, 3.37)	**0.046**
Model 2	2.13 (1.00, 4.42)	**0.046**	1.83 (0.96, 3.42)	0.061
Model 3	1.97 (0.92, 4.11)	0.074	1.75 (0.92, 3.29)	0.082
**Hospital stay (3 years)**				
Model 1	0.88 (0.52, 1.46)	0.640	0.78 (0.51, 1.19)	0.259
Model 2	0.86 (0.50, 1.43)	0.563	0.74 (0.47, 1.13)	0.167
Model 3	0.85 (0.49, 1.44)	0.551	0.64 (0.41, 1.00)	0.056
**Hospital stay (7 years)**				
Model 1	1.14 (0.59, 2.12)	0.693	0.75 (0.43, 1.26)	0.288
Model 2	1.06 (0.54, 2.00)	0.863	0.73 (0.42, 1.24)	0.262
Model 3	1.04 (0.52, 1.99)	0.907	0.72 (0.41, 1.23)	0.240
**Nursing care (3 years)**				
Model 1	2.44 (1.14, 5.01)	**0.018**	3.94 (2.00, 7.76)	**<0.001**
Model 2	2.05 (0.93, 4.32)	0.065	3.41 (1.69, 6.83)	**0.001**
Model 3	2.43 (1.08, 5.27)	**0.028**	2.95 (1.44, 5.99)	**0.003**
**Nursing care (7 years)**				
Model 1	2.68 (1.26, 5.53)	**0.009**	1.84 (0.95, 3.49)	0.066
Model 2	2.74 (1.26, 5.80)	**0.009**	1.80 (0.91, 3.49)	0.085
Model 3	2.65 (1.20, 5.67)	**0.013**	1.72 (0.86, 3.34)	0.115
**Disability (3 years)**				
Model 1	3.07 (1.64, 6.22)	**0.001**	1.50 (0.99, 2.32)	0.062
Model 2	2.70 (1.42, 5.53)	**0.004**	1.48 (0.95, 2.33)	0.084
Model 3	2.46 (1.27, 5.12)	**0.011**	1.32 (0.84, 2.11)	0.234
**Disability (7 years)**				
Model 1	2.03 (0.91, 5.07)	0.102	2.21 (1.25, 4.05)	**0.008**
Model 2	1.73 (0.75, 4.37)	0.218	2.30 (1.27, 4.28)	**0.007**
Model 3	1.37 (0.57, 3.63)	0.500	2.22 (1.21, 4.22)	**0.012**
**Multimorbidity (3 years)**				
Model 1	2.08 (1.13, 4.14)	**0.026**	1.38 (0.88, 2.19)	0.166
Model 2	1.91 (1.03, 3.82)	0.052	1.39 (0.88, 2.22)	0.164
Model 3	1.88 (0.99, 3.84)	0.066	1.25 (0.78, 2.03)	0.367
**Multimorbidity (7 years)**				
Model 1	1.19 (0.57, 2.71)	0.658	1.43 (0.81, 2.63)	0.234
Model 2	1.08 (0.52, 2.50)	0.838	1.41 (0.79, 2.61)	0.258
Model 3	0.86 (0.39, 2.06)	0.724	1.42 (0.78, 2.68)	0.264

ADL: activities of daily living; CI: confidence interval; OR: odds ratio.

Model adjustment:
Model 1: Age, sex.
Model 2: Model 1 + education, physical activity, nutrition, sleep duration, alcohol consumption.
Model 3: Model 2 + albumin, arthritis, neurological disease, lung disease, polypharmacy.

Bold font for p-values indicates significance ($p \leq 0.05$).

References

1. R Core Team. A Language and Environment for Statistical Computing. R Foundation for Statistical Computing, Vienna, Austria. https://www.R-project.org/. 2023.

2. Larsson J. eulerr: Area-Proportional Euler and Venn Diagrams with Ellipses. R package version 7.0.2, https://CRAN.R-project.org/package=eulerr. 2024.

3. Therneau T. A Package for Survival Analysis in R. R package version 3.7-0, https://CRAN.R-project.org/package=survival. 2024.
